# Power Frequency Magnetic Fields Affect the p38 MAPK-Mediated Regulation of NB69 Cell Proliferation Implication of Free Radicals

**DOI:** 10.3390/ijms17040510

**Published:** 2016-04-06

**Authors:** María Antonia Martínez, Alejandro Úbeda, Jorge Moreno, María Ángeles Trillo

**Affiliations:** 1 Servicio de Investigación-BEM, University Hospital Ramón y Cajal-IRYCIS, 28034 Madrid, Spain; axumaeso@gmail.com (A.U.); angeles.trillo@hrc.es (M.Á.T.); 2 Departamento de Ingeniería Eléctrica, Electrónica y de Automatización y Física Aplicada, Technical School of Engineering and Industrial Design (ETSID), UPM, 28012 Madrid, Spain; jorge.moreno@upm.es

**Keywords:** extremely low frequency, magnetic field, neuroblastoma, cell cycle, NAC, ERK1/2, JNK

## Abstract

The proliferative response of the neuroblastoma line NB69 to a 100 µT, 50 Hz magnetic field (MF) has been shown mediated by activation of the MAPK-ERK1/2 pathway. This work investigates the MF effect on the cell cycle of NB69, the participation of p38 and c-Jun N-terminal (JNK) kinases in the field-induced proliferative response and the potential involvement of reactive oxygen species (ROS) in the activation of the MAPK-ERK1/2 and -p38 signaling pathways. NB69 cultures were exposed to the 100 µT MF, either intermittently for 24, 42 or 63 h, or continuously for periods of 15 to 120 min, in the presence or absence of p38 or JNK inhibitors: SB203580 and SP600125, respectively. Antioxidant *N*-acetylcysteine (NAC) was used as ROS scavenger. Field exposure induced transient activation of p38, JNK and ERK1/2. The MF proliferative effect, which was mediated by changes in the cell cycle, was blocked by the p38 inhibitor, but not by the JNK inhibitor. NAC blocked the field effects on cell proliferation and p38 activation, but not those on ERK1/2 activation. The MF-induced proliferative effects are exerted through sequential upregulation of MAPK-p38 and -ERK1/2 activation, and they are likely mediated by a ROS-dependent activation of p38.

## 1. Introduction

A number of epidemiological studies have reported on potential association between exposure to power frequency (50–60 Hz) magnetic fields (MF) and increased risk for various diseases, including amyotrophic lateral sclerosis, brain tumors or childhood and adult leukemia [1,2,3,4,5,6]. Based primarily on the epidemiology of childhood leukemia, the International Agency for Research on Cancer (IARC) included the extremely low frequency (ELF: 3 Hz–3 kHz) MF in its records of possible carcinogens for humans (group 2B) [7]. There is experimental evidence *in vitro* that provides partial support to the epidemiological data, as it reveals that ELF MF can affect different cellular events involved in cancer processes [8]. Nevertheless, the fact that the basic interaction mechanisms of weak MF with biological systems have not been satisfactorily identified so far, has contributed to the existing uncertainty about the potential health effects of these fields.

Since the energy transmitted by low frequency magnetic or electromagnetic fields is not high enough to affect chemical bonds, it is generally accepted that ELF fields are not capable of damaging the DNA directly [9]. Nevertheless, a number of studies have reported on changes on DNA integrity after exposure to 50 Hz MF at flux densities ranking 0.1–1 mT [10,11,12] and several hypotheses have been put forward on how ELF fields might indirectly affect DNA structure [13,14,15]. For instance, it has been proposed that if MF exert a tumorigenic effect, they would do through tumor growth promotion, involving, among other mechanisms, a mitogenic-induced signal transduction that causes the cell to proliferate. In fact, on the basis of the block of experimental studies that have revealed proliferative responses in different *in vitro* systems exposed to 50/60 Hz MF within the 10–5000 µT rank, it has been proposed that such effects would involve signaling events at the cell membrane level, as well as subsequent processes of signal amplification [16,17,18,19]. For instance, it has been reported that in different cell types, including INIT/C3H/10T1/2 mouse fibroblasts, 3Y1 rat fibroblasts, CHL hamster lung cells, HL-60 human leukemia cells and MCF-7 human breast cancer, exposure to 50/60 Hz MF induces changes in signal transduction pathways that are known to be directly involved in proliferative processes [20,21,22,23]. Those signaling pathways, such as the Mitogen-Activated Protein Kinases—Extracellular-Signal-Regulated Kinase 1 and 2 (MAPK-ERK1/2), MAPK-p38 and -c-Jun N-terminal (JNK), also respond to chemical or physical stressors such as cytokines, UV radiation or heat shock [24]. Moreover, it has been shown that prolonged exposure of neuroblastoma cells to a 50 Hz, 1 mT MF, besides increasing their proliferative activity, induces dramatic changes in the proteomic profile, particularly in proteins whose overexpression is related to high malignant potential, drug resistance, cytoskeleton re-arrangement and enhanced defense against oxidative stress [25]. Nevertheless, the *in vitro* results as a whole are somewhat inconsistent, since different studies have reported a variety of responses, including proliferative, antiproliferative or differentiating effects, as well as lack of effect [16,17,18,19,20,21,22,23,26,27,28]. It has been proposed that such variability could be due in part to the involvement of a number of physical (signal wave form, magnetic flux density, exposure time, exposure cycle) and biological factors (cell type, genetics and/or physiology) that would be critical to the cellular response [8].

Previous studies by our group have shown that 50 Hz MF at 10 or 100 µT induce increased DNA synthesis and cell proliferation in two human cancer cell lines: hepatocellular carcinoma HepG2 and neuroblastoma NB69 [18,29,30,31,32]. In order to investigate whether such responses might be mediated by field-induced changes in cell cycle regulation, the present work analyzes through flow cytometry the cell cycle of NB69 exposed intermittently to the MF for 24, 42 or 63 h, as well as the effects of short-term exposure (120 min) on the expression of cyclin D1 and p27, two key proteins in cell cycle regulation. Cyclin D1 regulates the progression of the G1 phase and its transition to S phase, and its expression is sensitive to a number of environmental factors [33]. As this cyclin becomes activated by binding to cyclin-dependent kinases (CDK), the present study also investigates the potential influence of the MF on the expression of p27Kip, a cyclin-dependent kinase inhibitor. This protein suppresses the activity of cyclin-CDK complexes, which leads to blockage of the cell cycle between phases G1 and S. Consequently, the decreased expression or deficiency of p27 strongly correlates with poor prognosis in multiple cancer types [34].

We have also reported that changes in MAP-ERK1/2 activation are involved in MF-induced functional alterations in NB69 proliferation [31]. The present study investigates the possibility that other signaling pathways relevant to cell proliferation and survival, such as MAPK-JNK and p38 [35,36], are also involved in the proliferative response of NB69 to the MF.

In addition, evidence exists supporting the hypothesis that alterations in the regulation and/or generation of reactive oxygen species (ROS) free radicals (FR) could be among the main causes of the effects induced by ELF MF on different biological systems [37]. Furthermore, it has been shown that free radicals can be involved in the proliferative response and DNA damage induced by *in vivo* or *in vitro* exposure to weak (0.5 µT–1.0 mT) 50 Hz MF [12,17,38]. In the present study the radical scavenger *N*-acetyl-l-cysteine (NAC) was used as a tool for investigating the potential involvement of ROS on the proliferative effects of the MF on NB69.

The results show that exposure to a 50 Hz, 100 µT MF causes changes in the regulation and kinetics of the NB69 cell cycle, which leads to significant alterations in the regulation of cell proliferation and cell cycle progression, as reported in the present and previous studies [18,31,32]. In fact, and in consonance with our published results on the MF effects on MAPK-ERK1/2, here we show that the field exposure causes transient activation of the MAPK-p38 pathway, which has revealed being involved in the MF proliferative effects. Indeed, the present results show that the proliferative response to the MF can be blocked by pharmacological inhibition of p38, but not by JNK inhibition. Furthermore, NAC can block the field effects on cell proliferation and p38 activation, but not those on ERK1/2 activation.

## 2. Results

### 2.1. Effects of the 100 µT Magnetic Fields (MF) on the Cell Cycle

**Figure 1:**
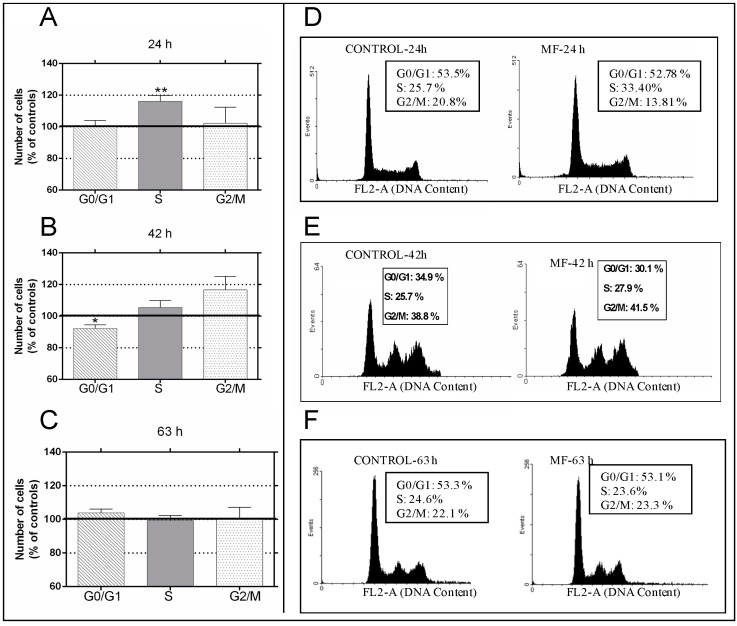
Magnetic field (MF) (50 Hz, 100 µT) effects on cell cycle progression. Samples exposed to MF for 24 h (**A**) 42 h (**B**) or 63 h (**C**) were flow cytometry analyzed after propidium iodide staining. Percentages of diploid cells (DNA content) at G0/G1, S and G2-M phases were determined using CellQuest 3.2 software (BD Biosciences, San Jose, CA, USA). The data, normalized over controls in the corresponding phases (line 100%), are means ± SEM of six experimental runs per time interval. Statistically significant differences with respect to controls: 0.01 ≤ * *p* < 0.05; 0.001 ≤ ** *p* < 0.01 (Student’s *t*-test.) (**D**–**F**): Representative cell cycle profiles in sham-exposed controls (left) and samples exposed to the MF (right) for 24, 42 or 63 h, respectively.

**Figure 2:**
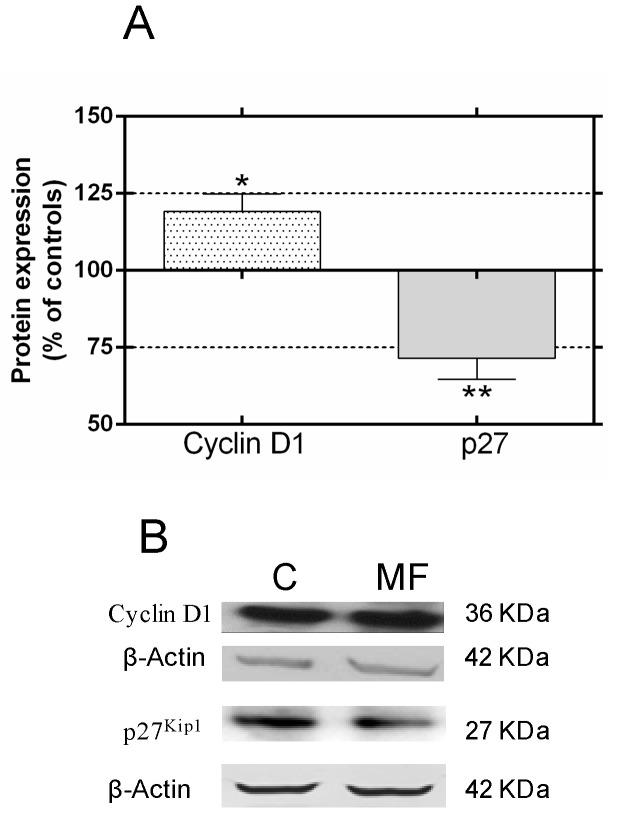
Field effects on cell cycle regulatory proteins. (**A**) Expression of cyclin D1 and p27^kip1^ after 120 min of MF- or sham-exposure, using β-actin as loading control. The data, normalized over the respective values of protein expression in sham-exposed controls (line 100%), are means ± SEM of six experimental replicates. Statistically significant differences with respect to controls: 0.01 ≤ * *p* < 0.05; 0.001 ≤ ** *p* < 0.01 (Student’s *t*-test); (**B**) Representative blots of cyclin D1, p27^kip1^ and β-actin expression.

As revealed by flow cytometry, MF exposure significantly changed the distribution of the cell cycle phases (Figure 1). In samples exposed for 24 h (Figure 1A,D), consistent significant changes were observed in the rate of cells in S phase (16.1% increase above controls; *p* < 0.01). The 42-h exposure (Figure 1B,E) caused significant decline in the rate of cells in G0/G1 (7.8% below controls, *p* < 0.05), accompanied by slight increases in the rates of cells in phases S and G2/M (5.5% and 16.6% above controls, respectively). Taken separately, these increases were not statistically significant, but they were when both phases are considered together: S + G2/M (8.7% ± 1.99% above controls; *p* < 0.01). As could be expected, these changes in the cell cycle were no longer detectable in later stages, after 63 h of incubation and MF exposure (Figure 1C,F), since at such later stages the cultures stabilize due to the chosen experimental conditions, which do not involve periodic renewal of the media. Western blotting analysis of the expression of the cell cycle regulatory proteins (Figure 2A,B) revealed that short-term field exposure (120 min) significantly increased the expression of cyclin D1 (19.1% above controls; *p* < 0.05) and decreased that of p27 (28.5% below controls; *p* < 0.01). It is likely that these changes in the early expression of cell cycle control proteins would lead to the observed increased proportion of cells in the S-phase at 24 h, as well as to a blockade or slowing down of phase G1 at 42 h, accompanied with increased rates of cell in S + G2/M.

### 2.2. Effects of p38 and JNK Inhibition on the MF-Induced Proliferative Response

**Figure 3:**
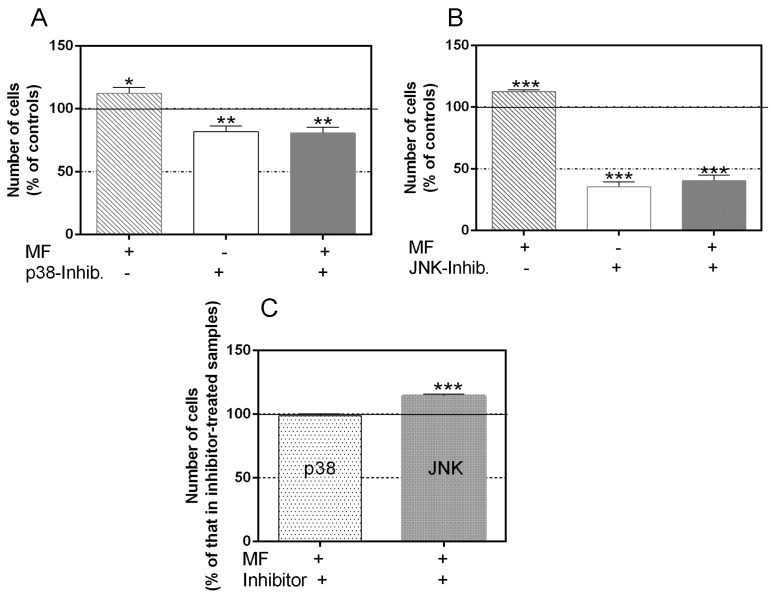
Cell response to 63 h of MF exposure in the presence or absence of MAPK-p38 and -JNK inhibitors. (**A**) Number of viable cells, determined by Trypan blue exclusion assay, after MF exposure and/or p38-inhibitor treatment. The data, normalized over the respective sham-exposed, untreated controls (line 100%), are means ± SEM of four experimental replicates. Statistically significant differences with respect to controls: 0.01 ≤ * *p* < 0.05; 0.001 ≤ ** *p* < 0.01 (ANOVA followed by the Student’s *t*-test.); (**B**) Number of viable cells after MF exposure and/or JNK-inhibitor treatment. The data, normalized over the respective sham-exposed, untreated controls (line 100%), are means ± SEM of four experimental replicates. Statistically significant differences with respect to controls: *** *p* < 0.001 (ANOVA followed by the Student’s *t*-test.); (**C**) Cell number after MF exposure in the presence of p38- or JNK- inhibitors. The data, normalized over those in samples sham-exposed in the presence of p38 or JNK inhibitors, are means ± SEM of four experimental replicates. Statistically significant differences with respect to the corresponding inhibitor-treated controls: *** *p* < 0.001 (Student’s *t*-test).

As shown in Figure 3A, 63 h of intermittent exposure to the MF induced a significant increase in cell number (12.4% above sham-exposed controls), confirming the previously reported proliferative response of NB69 to MF exposure [18,31,32]. Administered alone, the p38 inhibitor SB203580 (SB) resulted in a significant decrease in cell number (81.7% of that in controls; *p* < 0.01, Figure 3A), which was of the same order as that obtained when the inhibitor was applied in the presence of the MF (80.7% of that in controls; *p* < 0.01). Thus, the cellular response to the combined treatment did not differ from that of the controls that were sham-exposed to the MF and treated with p38 inhibitor (Figure 3C). In both groups, the observed decrease in cell population with respect to untreated controls can be attributed to reduced cell proliferation, as none of the treatments affected the cell viability of the cultures (data not shown). The fact that the p38 inhibitor blocked the proliferative response to the field indicates that the MAPK-p38 pathway is involved in such a response. As for the treatment with the JNK inhibitor SP600125, when administered alone it resulted in a significantly decreased cell number (35.5% of that in untreated controls, *p* < 0.001; Figure 3B). This decrease was slightly but significantly less pronounced when the inhibitor was administered in the presence of the field (40.6% of that in controls, *p* < 0.001). In fact, when in the presence of the JNK inhibitor, the cell number in MF-exposed samples was significantly higher (14.4%, *p* < 0.001) than in the sham-exposed ones (Figure 3C), indicating that the MF-induced proliferative response would not be mediated by activation of the JNK pathway.

### 2.3. MF Exposure Induces Early Activation of p38 and JNK

**Figure 4:**
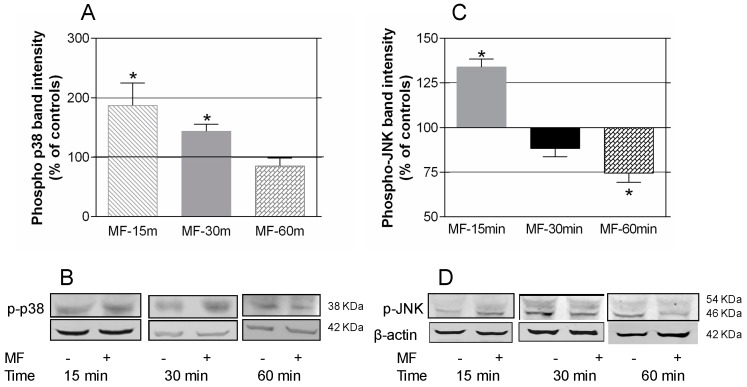
Western blotting quantification of MAPK-p-p38 and -p-JNK protein expression after short-term exposure to the MF. (**A**) Expression of phosphorylated-p38 after 15, 30 or 60 min of MF- or sham-exposure. The data, normalized over the corresponding controls (line 100%), are means ± SEM of at least four experimental replicates. Statistically significant differences with respect to controls: 0.01 ≤ * *p* < 0.05 (Student’s *t*-test.); (**B**) Representative blots for p-p38 after MF- or sham-exposure, using β-actin as loading control; (**C**) Expression of phosphorylated-JNK. Means ± SEM of four experimental repeats, normalized over controls (line 100%) Statistically significant differences with respect to controls: 0.01 ≤ * *p* < 0.05 (Student’s *t*-test.); (**D**) Representative blots at the different MF- or sham-exposure intervals. The p-JNK band is the one showing a 46 KDa molecular weight.

Western blotting was used to further investigate the potential involvement of the MAPK-p38 and -JNK pathways in the MF effects. As shown in Figure 4A,B, shortly after the exposure onset, the field induced increased expression of phospho-p38 (p-p38). Indeed, at 15 min of MF exposure, the effect seemed to be stronger (87.3% over controls; *p* < 0.05) than at 30 min (43.8% over the corresponding controls; *p* < 0.05), whereas after 60 min of exposure, the p-p38 expression levels did not differ from those in sham-exposed controls. The levels of phospho-JNK (p-JNK) expression (Figure 4C,D) were also increased after 15 min of MF exposure (33.83% ± 4.55% over controls; *p* < 0.01). However, the effect was reversed soon after, reaching a significant decrease in p-JNK expression at 60 min of exposure (25.42% ± 5.3% below the corresponding controls; *p* < 0.01).

**Figure 5:**
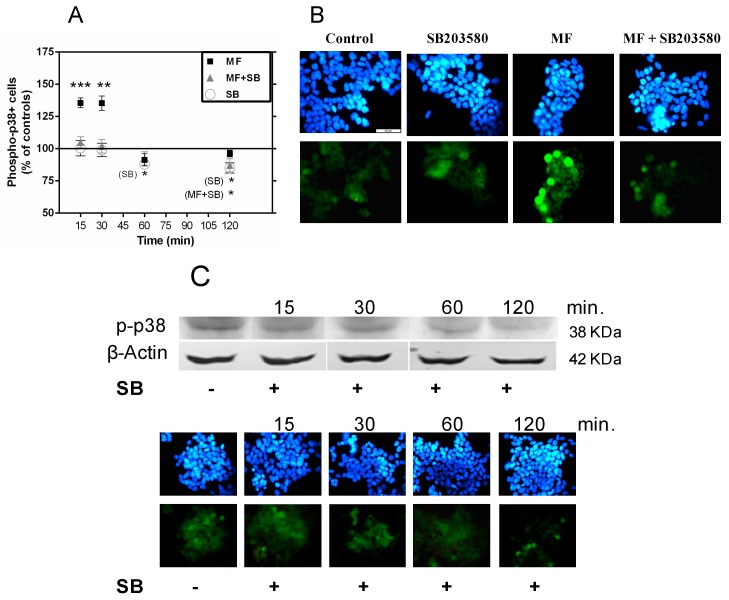
Quantification of phospho-p38-labeled cells after short-term MF exposure. (**A**) The data, obtained by computer-assisted analysis of immunocytochemical images, are normalized over the respective controls. Each point corresponds to the mean ± SEM of four experimental replicates. Four conditions were tested: sham-exposure of untreated controls (line 100%), MF exposure only, and p-p38 inhibitor SB203580 treatment, alone (SB) or combined with MF (MF + SB). Statistically significant differences with respect to controls: 0.01 ≤ * *p* < 0.05; 0.001 ≤ ** *p* < 0.01; *** *p* < 0.001 (ANOVA and Student’s *t*-test); (**B**) Representative images of immunocytochemical staining for phospho-p38 (**lower** panels) in controls, in cells exposed to MF for 15 min, in samples treated with the inhibitor onlyand in samples treated with MF + SB203580 for 15 min. All cell nuclei were stained with Hoechst (**upper** panels); (**C**) The specificity of SB203580 as MAPK-p38 inhibitor was assessed by p38 phosphorylation assay, showing that 10 µM inhibitor induced a time-dependent reduction of the spontaneous activation of p38. Representative images of immunoblots (**upper** panel) and immunocytochemistry (**lower** panel). Scale bar = 50 µm.

The study of pathways potentially involved in the proliferative response to the MF was extended by immunocytochemical analysis of p-p38 expression. The potential field effects on JNK phosphorilation were not immunofluorescence tested because the data show that the MF-induced increase in cell number is blocked by the inhibition of p38, but not by JNK inhibition. The results in Figure 5A,B show that short, 15 or 30 min exposure to MF significantly increased the number of phospho-p38 labeled cells (p-p38+ cells, 35.6%, *p* < 0.001 and 35.3%, *p* < 0.01, respectively, over the corresponding controls), whereas longer exposure intervals, of 60 and 120 min, did not change significantly the percentages of p-p38+ cells with respect to sham-exposed controls. As for the p38 inhibitor, when administered alone for 15 or 30 min, it did not affect the number of p-p38+ cells, but blocked the response to the field at these intervals. However, at 60 min, the p38 inhibitor alone induced significant decrease in the number of p-p38+ cells (88.02% of that in controls *p* < 0.05). After 120 min of treatment with the inhibitor, alone or in the presence of MF, the number of cells expressing p-p38 was significantly reduced when compared to controls treated with vehicle (85.06% and 87.14% of that in controls, respectively, *p* < 0.05). These results were supportive of those obtained by immunoblot. The specificity of SB203580 as MAPK-p38 inhibitor was assessed by p38 phosphorylation assays (immunoblots, and immunocytochemistry) showing that 10 µM inhibitor induced a time-dependent reduction of the spontaneous activation of p38, (Figure 5C).

### 2.4. N-Acetylcysteine Blocks the MF Proliferative Effects

**Figure 6:**
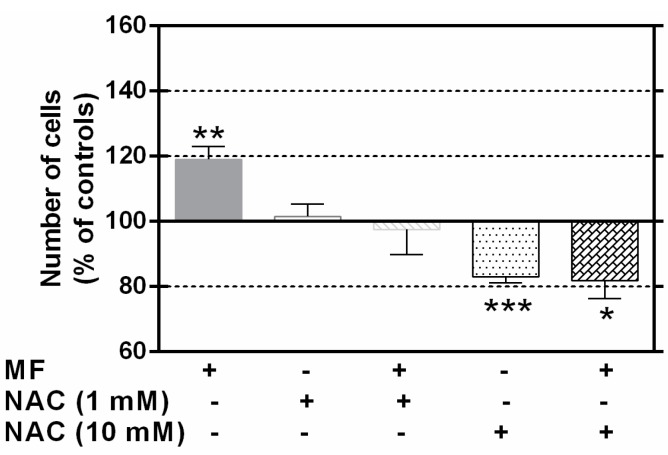
Number of viable cells after 63 h of intermittent exposure to the MF, in the presence of NAC 0.0 (vehicle), 1.0 or 10.0 mM. The data, normalized over the corresponding controls, are means ± SEM of four experimental replicates. Statistically significant differences with respect to controls: 0.01 ≤ * *p* < 0.05; 0.001 ≤ ** *p* < 0.01; *** *p* < 0.001 (ANOVA and Student’s *t*-test).

The potential involvement of free radicals in the proliferative response to the MF was investigated by exposing the cells for 63 h in the presence or absence of the chelating agent NAC. At a dose of 1 mM, NAC completely blocked the proliferative effect of the field (Figure 6), whereas at a concentration one order of magnitude higher, NAC caused significant reduction in cell number (approximately 17% below the corresponding controls), both under MF- and sham-exposure conditions. These effects were not attributable to decreased average cell viability, which was 79.6% in controls, *vs* 78% and 81.4% in samples treated with 1.0 and 10.0 mM NAC, respectively (data not shown). These survival rates correspond to those expected in NB69 cells grown in standard conditions [39].

### 2.5. N-Acetylcysteine Blocks the MF-Induced Activation of p38 But Not ERK1/2 Activation

**Figure 7:**
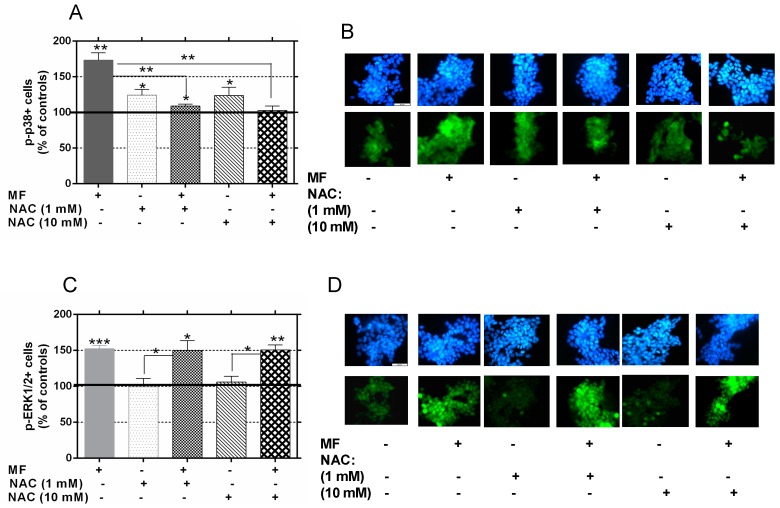
Expression of p-p38 and p-ERK1/2 after 30-minute MF exposure in the presence of 1 or 10 mM NAC. Immunocytochemistry and computer-assisted image analysis. (**A**) Phospho-p38 positive cells. The data, normalized over sham-exposed controls treated with NAC vehicle (line 100%), are means ± SEM of four replicates per experimental condition. Statistically significant differences with respect to controls: 0.01 ≤ * *p* < 0.05; 0.001 ≤ ** *p* < 0.01; *** *p* < 0.001 (ANOVA and Student’s *t*-test); (**B**) Representative images of immunocytochemical staining for p-p38 (lower panels) in control, NAC (1 or 10 mM), MF exposed or MF + NAC samples. Cell nuclei (upper panels) were Hoechst stained; (**C**) Phospho-ERK1/2 positive cells. The data, normalized over controls (line 100%), are means ± SEM of four replicates per experimental condition. Statistically significant differences with respect to controls: 0.01 ≤ * *p* < 0.05; 0.001 ≤ ** *p* < 0.01; *** *p* < 0.001(ANOVA and Student’s *t*-test); (**D**) Representative images of immunocytochemical staining for p-ERK1/2; same notations as in (**B**). Scale bar = 50 µm.

On the basis of the above results on p38 activation, and of those showing that 30 min of MF exposure can activate ERK1/2 [31], a preliminary study was conducted to investigate the potential involvement of free radicals in the MF-induced activation of p38 and ERK1/2 pathways. A study of JNK phosphorylation was not carried out since, as stated above, the present data show it is unlikely that the JNK pathway is involved in the proliferative effect of the MF. The early expressions of p-p38 and phospho-ERK1/2 (p-ERK1/2) were analyzed in cells exposed to the MF for 30 min in the presence or absence of 1 or 10 mM NAC. The results illustrated in Figure 7 show statistically significant increases in the rates of p-p38+ (Figure 7A,B) and p-ERK1/2+ cells (Figure 7C,D) after MF exposure in the absence of NAC (73.4% and 52.3% above sham-exposed controls, respectively). Under sham-exposure conditions, NAC also induced significant increases in the rate of p-p38+ cells at concentrations of 1 or 10 mM (24.3% and 28.8% above controls, respectively, Figure 7A). These increases were attenuated by the field exposure, having the cultures exposed to the MF in the presence of 10 mM NAC showed an average rate of p-p38+ cells equivalent to that in sham-exposed controls. Taken together, these data show that free radical chelation by NAC can block, potentially in a dose-dependent manner, the MF-induced activation of p38. As for the rate of p-ERK1/2+ cells (Figure 7C,D), it was not affected by any of the two NAC concentrations when administered in sham-exposure conditions. Nor was the MF effect on p-ERK1/2 expression affected by simultaneous treatment with 1 or 10 mM NAC. Thus, the activity of the MAPK-ERK, as assessed by phosphorylated ERK, remained unchanged when cells were treated with magnetic field in presence or absence of NAC.

## 3. Discussion

The interest in the potential adverse effects of power frequency MF has been chiefly focused on the possibility that these fields may influence tumor promotion by increasing the rate of cell proliferation and/or modifying the activity of molecules involved in its regulation. However, the number of studies examining the proliferative responses to magnetic flux densities below the 1000 µT threshold for workers’ protection against 50 Hz MF [40] is rather scarce [17,31,32,41,42]. To investigate the molecular mechanisms involved in the proliferative response of human tumor cells to weak MF, we have exposed cultures of the neuroblastoma line NB69 to a 50 Hz, 100 µT field.

The results show an average cell number increase of approximately 15% over controls after 63 h of intermittent exposure, which confirms our previously reported data, at the same time or earlier time points (24, 42 and 63 h), that the MF promotes proliferation and/or DNA synthesis in human cancer cells [18,31,32]. Increased proliferation was paralleled by significant increase in the percent of cells in the S phase, which reached a maximum of 16% over controls after 24 h of intermittent exposure. This was followed by significantly decreased proportion of cells in the G0–G1 phase and increased percent of cells in the S+G2/M at 42 h. These effects are consistent with those reported by our group [18,31] and by others [17,25,43] on MF-induced increased proliferation rates and/or DNA synthesis, and indicate that exposure to weak 50 Hz fields can exert mitogenic effects on neuroblastoma cells. Western blotting analysis of the expression of cell cycle regulatory proteins revealed that a short (120 min) field exposure caused statistically significant increase in cyclin D1 expression (19.1% above controls) and a decrease in p27 expression (28.5% below controls). Taken together, these results suggest that the MF causes significant changes in cell cycle progression that will later result in cell number increase, as observed after 63 h of incubation and field exposure in this and in a previous work [31]. Ongoing research will investigate the field effects on mRNA levels of cyclin D1 and p-27 genes. Also the potential effects induced by the MF on these cyclins at the time intervals of 24 or 42 h, where significant changes were observable in the cell cycle remain to be investigated.

We have reported in previous studies that the MAPK-ERK1/2 signaling pathway is involved in the proliferative effects of the MF in NB69, since the 3 h On/3 h Off intermittent treatment induced early, transient and cyclic activation of ERK1/2, which peaked at 30 min of the “On” intervals of field exposure [31]. The present results confirm such ERK1/2 activation and show that the MF also activates the MAPK-p38 pathway in a time-dependent manner, similar to that through which the field modifies the activation pattern of p-ERK1/2. This suggests that MAPK-p38 can be involved in the field-induced growth response.

Our previously published work also showed that at early time points (30 or 60 min) treatment with p-ERK inhibitor inhibits MF-induced ERK phosphorylation, but does not inhibit ERK phosphorylation itself [31]. However, at 120 min the pERK inhibitor alone induced a significant decrease in the number of p-pERK+ cells. A similar response is described herein using p38 inhibitor SB203580 (at early time points of 15−30 min), which indicates that the inhibitor acts gradually with time, but does not rule out the possibility that the inhibitor is active relatively soon after MF exposure onset. It has been reported that SB strongly inhibits the activity of p38 MAPK as it inhibits activation of MAPKAPK2, a specific physiological substrate of p38 MAPK. In fact, SB acts by blocking the p38 kinase MAPKAPK2 cascade, forming a p38+SB-complex [44]. These changes in the protein conformation prevent ATP binding, which subsequently prevents activation of p38. Therefore, it is possible that at early exposure time, SB induces conformational changes and blocks ATP binding, thereby inhibiting the MF effect without noticeably affecting p38 phosphorylation in the short term. The results reported herein also show that the MF causes early and transient activation of the JNK pathway at a magnetic flux density weaker than those applied in other studies (400 and 800 µT) using mouse cells [22]. However, such activation does not seem to be involved in the proliferative response induced by the MF in NB69, since this response is not affected by pharmacological inhibition of the JNK pathway.

Additionally, the results show that:

(1) p38 activation increases shortly (15 min) after MF-exposure onset, which, when taken together with previously published data [31], indicates that activation of ERK1/2, detectable only at exposure times of 30 min or longer, is preceded by p38 activation; and

(2) activation of MAPK-p38 is required for effective growth stimulation of NB69, since pharmacological inhibition of this pathway reverted the proliferative response to the field at 63 h of intermittent exposure. This, taken together with previously reported data [31], reveals that activation of both ERK1/2 and p38 is critical to the MF-induced proliferative response, as it is suppressed by independent inhibition of either of the two pathways. However, the possibility cannot be ruled out that the observed field effects on the active forms of MAPK p38 and ERK1/2 are due to MF-induced increase in the expression of these proteins.

The magnitude and duration of the MAPK-p38 signaling activation are critical determinants of its biological effects [45,46]. Therefore, a prolonged activation of p38 has been related to apoptosis, whereas, in line with the present results, a transient activation has been associated with a proliferative response. In fact, over the past few years, members of the MAPK-p38 subfamily have joined the group of canonical signaling pathways involved in the transformation process [43]. Activation of p38 can also contribute to the epithelial-mesenchymal transition and to acquisition of invasive characteristics [47] and, ultimately, to tumor progression. For instance, MAPK-p38 displays robust induction in response to stressors such as UV and other carcinogenic radiations within the ionizing spectrum [48]. A proliferative effect similar to that reported in the present study, mediated by transient phosphorylation of the ERK1/2 and p38 pathways, has thus been described in normal human lung fibroblasts CCD-18Lu exposed to a low dose (0.05 Gy) of ionizing radiation [49]. However, only a few studies have reported activation of MAPK-p38 by power frequency MF [21] or have proposed that such activation is involved in the cellular response to these fields [50,51,52]. In general, these studies use magnetic flux densities well above the 100 µT tested herein, and describe antiproliferative responses and/or genic damage. For instance, Kim *et al.* [51] reported that repeated exposure (30 min a day for three consecutive days) to a 60 Hz MF at 6 mT increased p38 activation in normal (lung fibroblast IMR90) and cancer (cervical carcinoma HeLa) human cells. The effect was accompanied by increases in the frequency of DNA double-strand breaks and in the rate of apoptosis. Moreover, Wang and coworkers [52] reported that 24-h exposure to a 50 Hz, 0.2 mT MF causes p38 activation, along with a decrease in the proliferation of human foetal scleral fibroblasts. In contrast to this, our MF stimulus caused transient increase of p38 activation, accompanied by increased cell proliferation. These differences with respect to the other studies may be in part attributable to the fact that we applied a weak MF, with an intermittent exposure pattern.

On the other hand, it has been described that p38 promotes cell proliferation through ATF-2-mediated upregulation of cyclin D1 and cyclin A, and that it intervenes in the expression of cell cycle-regulating genes [53,54]. In addition, ERK is known to be directly implicated in the regulation of the G2/M transition, and a strict control of the kinetics and strength of ERK activation is necessary for proper progression of mitosis [55]. Besides, ERK1/2 phosphorylates H3, and such phosphorylation is hypothesized to intervene in the transcriptional activation of early genes through chromatin remodeling [56,57]. Therefore, it is possible that the herein reported activation of p38 and ERK1/2 can induce gene expression related to cell proliferation by regulating the cell cycle and remodeling the chromatin structure.

Moreover, one of the mechanisms suggested as involved in the biological effects of ELF MF is the generation and increased lifetime of FR [58,59] and it has been reported that FR can act as inducers of MAPK activation [60]. In this work, the study of the potential involvement of FR in the MF-induced proliferative response has been addressed through treatment with *N*-acetylcysteine, a tiol whose antioxidant, antigenotoxic and cancer-preventive properties have been well established [60,61,62]. NAC can easily enter the cell and, because of its -SH group, it acts as a ROS scavenger and is susceptible to deacetylation into cysteine, a relevant precursor in glutathione synthesis. This allows NAC to scavenge oxidants both directly and indirectly, for which it has been extensively used to study the role of ROS in different signaling pathways [63]. Our results show that 1.0 mM NAC, a concentration that does not affect proliferation in sham-exposed NB69, blocks the proliferative action of the MF, whereas a concentration 10 times higher equally inhibits the spontaneous proliferation in controls and that induced by the field. This reinforces the evidence that the NAC chemopreventive effects in cancer could be exerted through its nucleophilic, antioxidant activity [64]. Although the mechanisms through which 1.0 or 10.0 mM NAC blocks or inhibits, respectively, the proliferative action of the field remain to be elucidated, such NAC effects can be interpreted as indicative that ROS are involved in the MF-elicited cytoproliferative response.

The present results also show that, administered alone, NAC at 1.0 or 10.0 mM actually increases the cell fraction expressing p-p38, which may be due to the pro-oxidant effect of these NAC doses, as indicated by Swain and Faux [65]. This increase in the number of p-p38+ cells induced by NAC in sham-exposed conditions was of lesser magnitude than that induced by the MF in the absence of NAC. However, in the combined treatment, the activating action of the MF was significantly inhibited by NAC, returning the fraction of p-p38+ cells to values equivalent to those in controls.

**Figure 8:**
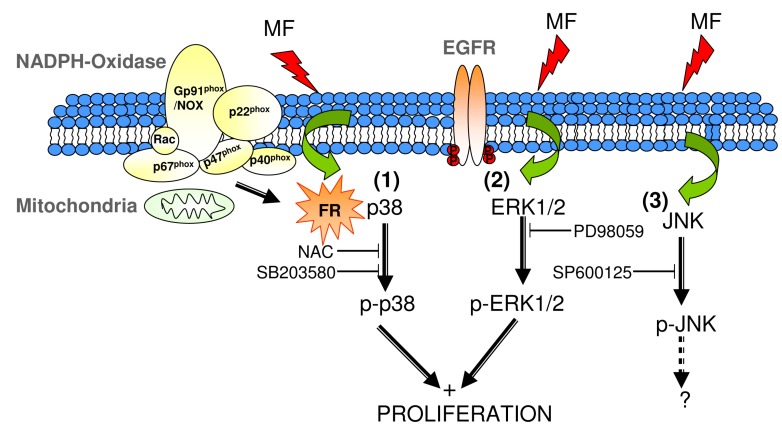
Schematic representation of potential molecular pathways and targets mediating the proliferative response to the MF, as from the present and previously published data. Proliferation would be induced through two different pathways: a FR-dependent one, mediated by early, transient activation of p38 (**1**) and an FR-independent pathway, mediated by transient activation of ERK1/2 (**2**). A proliferative response would trigger only when both pathways are activated. JNK activation induced by the MF would not intervene in the MF-proliferative effect (**3**).

It is conceivable that, as suggested by others, NAC can act either as an antioxidant or a pro-oxidant, depending on the experimental conditions [66,67]. Our results are consistent with those reported by Menon and coworkers [68] showing that NAC exerts an immediate (within 1 h) pro-oxidant effect, acting later on as an antioxidant agent. This view is consistent also with our results obtained by supplementing the medium with NAC 1 h prior to MF-or sham-exposure onset. Indeed, it can be postulated that the increased phosphorylation of p38 observed in controls at 90 min of sham-exposure would have occurred during the preceding 60 min of incubation with NAC. As for the effect of the MF exposure for the last 30 of the total 90 min of incubation with NAC, the antioxidant action of NAC would prevail and block the MF-induced promotion of p-p38 expression. Since NAC has elicited a pro-oxidative response in this study, the potential involvement of free radicals in the activation of p38 by the MF must be further investigated using different approaches. In any case, the fact that NAC inhibits field effects on p38 suggests that redox regulation of p38 could be a relevant mechanism mediating in the proliferative response to the MF. Besides, since the blocking of the proliferative effect by NAC does not interfere with the activation of ERK1/2 by the field, it is likely that the MF can activate both pathways through independent ways. And still, as mentioned before, the activation of both pathways has shown to be a determinant in the proliferative response of NB69. Taken together, these results corroborate the hypothesis that FR are involved in a number of reported effects of ELF MF [37], and provide support to the experimental results that link the proliferative effects of 50 Hz MF to ROS generation [17]. Also, MF-induced increases in FR have been shown to be related to mitochondrial alterations [69] or to changes in the enzymatic complex NADPH oxidase [70]. Although the present study has not investigated the FR source potentially affected by the MF, it can be proposed that involvement of the mitochondria or the NADPH oxidase in the potential increase of FR could be a cause for the herein reported MF-induced early activation of p38. Such activation would act as a triggering factor that, with the necessary contribution of the ERK1/2 activation, would result in the observed proliferative response to the MF (Figure 8).

In another vein, it has been reported that a blockade of the activation of the epidermal growth factor receptor (EGFR) inhibits cell proliferation in NB69 [71], and evidence exists that such a receptor is involved in MF-induced effects [72,73,74]. Furthermore, the MAPK-ERK1/2 pathway has been shown activated by EGFR [75], and MF exposure has been reported able to induce alterations in the tyrosine-kinase domain of EGFR [72]. Therefore, our results would be consistent with a potential involvement of EGFR in the MF-induced activation of ERK1/2 pathway (pathway 2 in Figure 8). In any case, the mechanisms proposed herein to understand the proliferative response do not rule out the possibility that the MF acted primarily on surface charges of the cell membrane [76] or that the proliferative effect were also mediated by MF-induced enhancement of the flux of Ca^2+^ ions through the membrane [69,76,77].

In sum, taken together, these data reveal that the proliferative effects induced in human neuroblastoma cells NB69 by intermittent exposure to a weak 50 Hz MF is mediated by, at least, two separate mechanisms. On the one side, the MF would cause early, transient and FR-dependent activation of the MAPK-p38 pathway. On the other hand, the MF also induces early and transient activation of the ERK1/2 pathway, through an FR-independent mechanism. Sequential and/or simultaneous MF activation of both mechanisms is necessary to induce a proliferative response in NB69, since blockade of any of the two pathways inhibits the proliferative response. These data contribute to deepening the knowledge on the molecular mechanisms underlying the potential mitogenic effects exerted by power frequency MF on cancer cells. It is obvious that from these results it cannot be directly inferred that weak power frequency MF are potentially harmful to humans. However, our data add to the growing pool of evidence indicating that the possibility that malignancy resulted from an accumulative effect of chronically repeated field exposure, cannot be ignored. The MF strength applied in the present study corresponds to 10%, 1.6% and 0.55% of the action levels for worker protection against sensorial or health effects of whole-body or limb exposure to 50 Hz MF, respectively [40]. Epidemiological and experimental evidence exists indicating that other human cell types can also respond to exposure to weak power frequency fields [1,2,8]. In any case, the available set of evidence allows for proposing that the limits established for protection of workers against immediate, harmful effects of short-term exposure to strong MF may not be useful for protection against frequent, longer or chronic occupational exposure. The study of the basic molecular mechanisms involved in the cellular response to weak MF is crucial for the development of effective strategies for protecting the public and workers against the potential, medium-to-long term adverse impact of certain parameters and conditions of field exposure.

## 4. Material and Methods

### 4.1. Cell Culture

The human neuroblastoma cell line NB69 was purchased from the European Collection of Authenticated Cell Culture (ECACC, Salisbury, UK). Cells were plated in 75 cm^2^ culture flasks containing Dulbecco’s Minimum Essential Medium (DMEM Biowhittaker, Lonza, Verviers, Belgium) supplemented with 15% heat-inactivated fetal bovine serum (FBS, Gibco BRL, Invitrogen, Paisley, Scotland, UK), 4 mM l-glutamine, 100 U/mL penicillin, 100 U/mL streptomycin and 0.25 µg/mL of amphotericin B as antimycotic agent (Gibco BRL, Invitrogen, Prat de Llobregat, Spain). Cultures were grown in an incubator (Forma Scientific, Thermo Fisher, Waltham, MA, USA) with a 37 °C, 5% CO_2_, humidified atmosphere. The cultures were trypsinized every seven days, part of the cells being subcultured in flasks and the remaining cells being seeded at a density of 4.5 × 10^4^ cells/mL, either directly on the bottom of 60 mm plastic Petri dishes (Nunc, LabClinics, Barcelona, Spain) or on coverslips placed inside the dishes. The medium was renewed immediately before MF exposure and/or chemical treatment.

### 4.2. Treatment with MAPK-p38 and -JNK Inhibitors

When needed, samples were treated with 10 µM of MAPK-p38 specific inhibitor, SB203580 [4-(4-fluorophenyl)-2-(4-methylsulfinylphenyl)-5-(4-pyridyl)-1H-imidazole] (BioSource, LabClinics, Barcelona, Spain). Also, 20 µM of SP600125 [Anthra(1,9-cd)pyrazol-6(2H)-one] (Biosource, LabClinics), a pharmacological inhibitor of c-Jun NH2-terminal kinase (JNK), was used. These concentrations have been proven effective in a number of studies [78,79]. The inhibitors were dissolved in dimethyl sulfoxide (DMSO; Sigma, Madrid, Spain). An equal dose of DMSO (vehicle) was added to the control dishes. The final DMSO concentration in the media was always ≤0.01%, which was proven not to influence the cell growth rate in the cultures (data not shown). On the third day after seeding (long-term proliferation assays) or on the fourth day after seeding (short-term assays), the medium was renewed and supplemented with the inhibitor or the vehicle 1 h prior to the MF- or sham-exposure onset.

### 4.3. Treatment with N-Acetyl-l-cysteine

*N*-acetyl-l-cysteine is an antioxidant containing an acetylated form of the amino acid l-cysteine that functions as a precursor on the synthesis of glutathione, a thiol involved in cellular detoxification [80,81]. The presence of sulfhydryl groups also enables NAC to neutralize free radicals. Thus, NAC is commonly used to inhibit reactive oxygen species and to identify and test inducers of these species. In a pilot test, three different doses of NAC (Sigma): 0.1, 1 and 10 mM, were assayed and compared to two control groups: one receiving and one not receiving the vehicle (deionized water). Three repeats, each with three dishes per experimental condition, were carried out. Only the samples treated with a 10 mM concentration of NAC responded with a reduction in cell number (data not shown). From these results, the doses of 1 and 10 mM were chosen for NAC treatment of the cultures following the same experimental procedure as that described above for p38 inhibitor.

### 4.4. Magnetic Field Exposure

The cultures were exposed to a 50 Hz, sine wave, vertically polarized MF, at a magnetic flux density (B_AC_) of 100 µT root mean square (rms). The MF exposure set-up has been described elsewhere [18,82]. Briefly, current flow was supplied by a wave generator (Newtronic Model 200MSTPC, Madrid, Spain) that has a 3.53 mA DC offset (B_DC_ of 15 µT rms). The generator was connected to a pair of coils set in Helmholtz configuration. Current in the coils was monitored using a multimeter (Hewlett Packard, model 974A, Loveland, CO, USA) and the induced MF was routinely checked by two magnetometers (EFA-3, Wandel and Goltermann, Eningen, Germany, and EMDEX II, Enertech Consultants, Campbell, CA, USA). One Helmholtz pair was placed inside each of two magnetically shielded chambers (Co-NETIC metal; Amuneal Corp., Philadelphia, PA, USA) located within two identical CO_2_ incubators (Forma Scientific, Thermofisher, Waltham, MA, USA). The background MF inside the shielded chambers was B_AC_: 0.04 ± 0.03 µT (rms); B_DC_: 0.05 ± 0.04 µT (rms). No increase of temperature at the sample location was observed using two pt100 thermocouple probes (Fluke, Model 52, Adler Instruments, Madrid, Spain) when the coils were energized to produce the desired magnetic flux density of 100 µT rms. In each experimental run, Petri dishes (five per experimental group) containing the cell samples were stacked in the central region of the Helmholtz coil gap, which ensured uniformity of MF exposure. Only one set of coils was energized in each experimental run. The samples in the unenergized set were considered sham-exposed controls. Following a random sequence, both coil sets and incubators were alternatively used for MF- or sham-exposure.

Deciphering the molecular basis of the initiation and progression of MF-induced proliferation, requires identification of key features and signal transduction pathways involved in such growth response. From there, the need of conducting both long- and short-term intervention studies testing the action of three agents: the MAPK-p38 and -JNK inhibitors, and *N*-acetyl-cysteine. Thus, for cell cycle and proliferation analysis, prolonged treatment (24–63 h) with intermittent MF exposure (3 h On/3 h Off cycles) was applied, which has proved effective in inducing changes in proliferation of the human neuroblastoma NB69 and hepatocarcinoma HepG2 cell lines [18,31]. For the study of the field effects on the early expression of proteins regulating intracellular signaling mechanisms, it was taken into account that after activation of the receptor, the response of expression/activation of the signal transduction pathways is relatively fast. Short treatments (15–120 min) with continuous exposure to the MF were therefore used. Following the same procedure as in our previous studies on ERK1/2 [31], these treatments were applied at day 4 postplating to ensure sufficient cell density to allow for correct analysis of the short-term response. A short exposure interval of 120 min was selected to investigate the effects on cyclin D1 and p-27 because the ERK pathway links mitogenic signals to the cell cycle machinery via regulation of cyclin D1 and the Cdk inhibitor p27. Initially, cyclin D1 expression requires activation of the Raf-Mek-Erk kinase cascade [83], which is activated by short-time exposure (30–60 min) to the MF [31]. The samples were analyzed at the end of the 120 min lapse of MF-or sham-exposure. As a whole, a total of 91 independent experimental runs, with more than 1000 samples (MF-exposed plus controls) were conducted in this study.

### 4.5. Analysis of Cell Cycle Progression

The cell cycle of MF-exposed cultures was analyzed by flow cytometry (FACScan Mod. FACScalibur, Becton Dickinson, Franklin Lakes, NJ, USA) and compared to that of the corresponding controls. After 24, 42, or 63 h of intermittent exposure, the cultures were trypsinized and both floating and adherent cells were collected. Samples were centrifuged at 1200 rpm for 5 min, fixed in 70% ethanol and stained with 3.4 mM sodium citrate, 20 µg/mL propidium iodide (Boehringer, Mannheim, Germany) and 100 µg/mL RNase A (Boehringer) solution. CellQuest 3.2 software was used for data acquisition (20,000 events per each of ten samples: five MF-exposed dishes *vs.* five sham-exposed controls, per experimental repeat) and analysis. Appropriate gating strategies were applied to exclude debris and aggregates.

### 4.6. Western Blotting

Western blotting assays were carried out to evaluate the expression of cell cycle control proteins cyclin D1 and p27Kip1. Briefly, four days after seeding, the cultures were MF- or sham-exposed, following the standard protocol. At 2 h of continuous MF-or sham-exposure, the proteins were extracted in hypotonic lysis buffer (10 mM Tris-HCL (pH 7.6), 100 mM potassium chloride (KCL), 1 mM EDTA, 1mM Ditiotreitol, 1 mM phenylmethylsulfonyl fluoride (PMSF), 10 µgr/mL Leupeptin, 5 µgr/mL Pepstatin A, 100 mM sodium fluoride (NaF), 20 mM β–glycerolphosphate, 20 mM sodium Molibdate, 0.5% Tritón X-100 and 0.1% SDS), and their concentrations were determined through Bradford’s assay [84]. The protein lysates (60 μg per lane) were resolved on 10% SDS/PAGE gels and transferred to nitrocellulose membranes (Hybond ECL, GE Healthcare, Little Chalfont, Buckinghamshire, UK). The membranes were blocked, and incubated overnight at 4 °C with mouse monoclonal antibodies against cyclin D1 (1:200 dilution; NCL-CYCLIN1-GM, Novocastra Laboratories, Newcastle upon Tyne, UK) or against p27Kip1 (1:500; AHZ0452, BioSource). β-Actin (1:5000; A5441, Sigma) was used as loading control. After washing, the membranes were incubated with horseradish peroxidase-labeled anti-mouse secondary antibody (GE Healthcare) for 1 h at room temperature. The bands were visualized by enhanced chemiluminescence (ECL) following the manufacturer’s instructions (GE Healthcare). The blots were analyzed by densitometric assay using PDI Quantity One-4.5.2 software (Bio-Rad, Hercules, CA, USA). Immunoblot assays were also conducted to evaluate the expression of phospho-p38 and phospho-JNK under MF- or sham-exposure, following the standard protocol. The cultures were MF- or sham-exposed for 15, 30 or 60 min intervals. Activation of p38 and JNK were detected with specific anti-phospho p38 (1:1000; 44–4846G, BioSource) and anti p-JNK (1:1000; 4668, Cell Signaling, Barcelona, Spain) antibodies. Also, for p-38, a horseradish peroxidase conjugated Anti-Rabbit IgG secondary antibody (1:5000; GE Healthcare), followed by ECL Advance Western Blotting Detection Kit (GE Healthcare) reaction was used. As for JNK, fluorescently labeled secondary antibody IRdye 800 CW, conjugated goat (polyclonal) anti-rabbit (1:10,000; LI-COR, Bioscience, LI-COR, Lincoln, USA) was used. The fluorescence signal was detected and quantified with Odyssey infrared imaging system (LI-COR). Equal loading of proteins was confirmed by β-actin (Sigma) immunoblot. For semiquantitative analysis of immunoblot bands, the optical density (OD) of each band was measured and quantified by densitometry through computer imaging (Quantity-One, Biorad, Munich, Germany).

At least four experimental replicates were conducted for each of the proteins studied. Three MF-exposed dishes *vs.* three sham-exposed dishes per experimental run and per exposure period were used.

### 4.7. Long-Term Exposure for Proliferation Assay. Growth Response at the End of a 63-h Lapse of Intermittent Exposure to MF in the Presence or Absence of p38 or JNK Inhibitors

Cell viability and proliferation were determined through Trypan blue (Sigma) exclusion and hemocytometer counting. The cells were seeded in 60 mm diameter plastic Petri dishes (Nunc) and cultured for three days. At that time the medium was renewed and the cultures were treated with the chemical inhibitor or with the matched vehicle (DMSO) concentration. Specifically, 20 Petri dishes were used in each of a total of four experimental runs. Two groups of five Petri dishes: five with p38 inhibitor and five with the vehicle, were distributed in each of the two coil sets placed inside the MF-shielded chambers. One hour later, the samples were cyclically exposed (3 h On/3 h Off) to the MF- or sham-exposed for a 63 h time lapse. A total of four different experimental conditions were assayed: (1) sham-exposure to the MF in the absence of inhibitor (untreated controls); (2) treatment with 10 µM p38 inhibitor only or 20 µM JNK inhibitor only; (3) MF exposure only; and (4) p38 or JNK inhibitor plus MF exposure. At the end of the 63 h interval the cells were harvested, collected in 1 mL of medium, stained with 200 µL of 0.04% Trypan blue in 0.6 mL Dulbecco’s PBS (GIBCO BRL, Invitrogen) and studied under a light microscope (Nikon TMS, Melville, NY, USA).

### 4.8. Long-Term Exposure for Proliferation Assay. Growth Response at the End of a 63-h Lapse of Intermittent Exposure to MF in the Presence or Absence of NAC

NB69 cells were plated on 30 Petri dishes: 10 dishes were treated with 1 mM NAC, 10 with 10 mM NAC and 10 with vehicle (deionized water). Sixty minutes after, the samples were distributed in each of the two sets of coils placed inside the MF-shielded chambers and submitted for 63 h to the following treatments: (1) sham-exposure in the absence of NAC (controls); (2) 1 mM NAC only; (3) 10 mM NAC only; (4) MF exposure only; (5) MF + 1 mM NAC; (6) MF + 10 mM NAC. In each of the four experimental replicates, five Petri dishes were used per experimental condition.

### 4.9. Immunocytochemistry Assay for Phospho-p38 Expression after Short-Term Exposure to MF

After four days of incubation according the above-described general procedures, except that the cells were seeded on 12 mm diameter coverslips placed into the Petri dishes, the cultures were MF- or sham-exposed for 15, 30, 60 or 120 min. The percent of phospho-p38 positive cells (p-p38+) was studied by immunocytochemistry. Immunostaining was performed by incubating the cells with primary antibody anti p-p38 (1:100, BioSource) and a secondary goat antirabbit IgG, antibody (1:500) conjugated with the fluorophore Alexa Fluor 488 (Molecular Probes, Invitrogen, Prat de Llobregat, Spain). The cell nuclei were counterstained with Hoechst 33342 (Bisbenzimide, Sigma). Images were captured with a fluorescence microscope (Nikon Eclipse TE300) and analyzed by computer imaging (Analy-SIS software, GMBH, Munich, Germany). In each of the four experimental runs, three coverslips were studied per experimental group: Sham-exposed controls, SB203580 or SP600125 inhibitor only, MF only, Inhibitor + MF. Fifteen microscope fields per coverslip were randomly selected for analysis. The total number of nuclei and the percent of p-p38 or p-JNK positive cells per microscope field were recorded.

### 4.10. Changes in the Expression of p-p38 and p-ERK1/2 in Response to Short-Term Exposure to MF in the Presence or Absence of NAC

At day 4 postplating, 10 samples were treated for 60 min with 1 or 10 mM NAC, or with vehicle (deionized water), and subsequently exposed or sham-exposed to MF for 30 min. This time lapse was chosen on the basis of previously reported results that ERK1/2 is significantly activated by a 30 min MF exposure [27]. Next, immunocytochemical analyses of p-p38 (1:100, BioSource) and p-ERK1/2 (1:100, 44–680G; BioSource) positive cells were conducted following the same protocol as described above.

### 4.11. Statistical Analysis

All experimental procedures and analysis were conducted blindly for treatment. Data were normalized and expressed as means ± standard error (SEM) of at least three independent experimental runs, using GraphPad Prism software (Graphpad Software, Inc., San Diego, CA, USA). The data corresponding to treated groups and their respective controls were compared by two-sample Student’s *t* test. Multifactorial one-way analysis of variance, ANOVA, was used to assess differences between multiple data sets. The limit of statistical significance was set at *p* < 0.05.
